# Cow and Ewe Cheeses Made with Saffron: Characterization of Bioactive Compounds and Their Antiproliferative Effect in Cervical Adenocarcinoma (HeLa) and Breast Cancer (MDA-MB-231) Cells

**DOI:** 10.3390/molecules27061995

**Published:** 2022-03-19

**Authors:** Mena Ritota, Raffaella Comitato, Pamela Manzi

**Affiliations:** Consiglio per la Ricerca in Agricoltura e L’analisi Dell’economia Agraria, Centro di Ricerca Alimenti e Nutrizione, Via Ardeatina 546, 00178 Rome, Italy; raffaella.comitato@crea.gov.it (R.C.); pamela.manzi@crea.gov.it (P.M.)

**Keywords:** cow and ewe cheese, saffron, bioactive compounds, crocin, safranal, picrocrocin, antiproliferative effect

## Abstract

Saffron is a widespread consumed spice containing many phytochemicals. It is often used in dairy technologies to enhance color and flavor of cheeses, but it is also known for its several therapeutic effects, as well as its antiproliferative and anticancer properties. In this study High Performance Liquid Chromatography was used to characterize saffron bioactive compounds in cow and ewe cheeses made with saffron, and the antiproliferative effect of the crocin-rich extracts from cheeses was investigated on different cellular lines (CaCo2, MDA-MB-231 and HeLa) by MTT assay. Crocins were observed in all cheese samples, with the total content ranging between 0.54 and 30.57 mg *trans*-4-GG/100 g cheese, according to the different cheese making process. Picrocrocin was detected in no cheese (probably due to its degradation during cheese making), while safranal was detected only in one ewe cheese (mainly due to its high volatility). HeLa and MDA-MB-231 cells were sensitive to treatment with crocin-rich extracts from cheeses, while no effect was observed on CaCo2 cells. The chemical environment of the food matrix seems to have a great influence on the crocin antiproliferative effect: the crocin-rich extracts from cheese with both high residual N/protein and fat contents showed increased antiproliferative effect compared to pure crocin (*trans*-4-GG), but cheeses from different milk species (type of fats and proteins) could also play an important role in modulating crocin’s antiproliferative effects.

## 1. Introduction

Italy plays an important role within the dairy industry worldwide, being one of the largest producers of typical PDO (Protected Designation of Origin) cheeses. Despite the wide range of dairy products, those that hold real importance in commerce are very few in number and often limited to some main cheeses such as Parmigiano Reggiano PDO and Grana Padano PDO. Ewe and goat milk cheeses, instead, are mostly produced on a small local scale, and they are not as competitive as cow milk cheeses. For this reason, cheese makers need to produce innovative and high-quality products, and to also meet the increasing demands of consumers.

Innovation in cheese has been in part driven by the addition of several ingredients, such as colorants, spices, leaves, flavors, and aroma agents. A common practice is the giving of a natural yellow to orange color to cheeses through the addition of spices: annatto, for example, is used in Cheddar and Mimolette cheese making. More recently, the inclusion of saffron in the cheese-making process is currently an increasingly practice as well.

Saffron, a perennial herb belonging to the *Iridaceae* family, has a long history as a culinary spice, but since ancient times it has been also used as medicinal plant [1]. In the food sector it is mainly known for its color, odor, and taste. Picrocrocin (4-(β-D-glucopyranosyloxy)-2,6,6-trimethyl-1-cyclohexene-1-carboxaldehyde), a monoterpene glycoside produced from zeaxanthin degradation, is responsible for the bitter taste of saffron, while its thermal or enzymatic degradation [2] gives rise to safranal (2,6,6-trimethylcyclohexa-1,3-diene-1-carbaldehyde), a monoterpenoid to which saffron aroma is mainly related. Safranal has also been revealed to show bear high antioxidant potential, as well as cytotoxicity towards certain cancer cells in vitro [3]. The yellow-red color of saffron is instead mainly due to a class of water-soluble carotenoids, crocins, which are esters of crocetin (8,8′-diapocarotenedioic acid) with different glycosidic moieties, such as glucose, gentiobiose, and neapolitanose. Further lipophilic carotenoids, such as lycopene, α-, and β-carotene, as well as zeaxanthin, phytoene, and phytofluene have also been reported in saffron in trace amounts [4]. In addition to its use as food coloring, crocin acts as an antioxidant by quenching free radicals, protecting cells and tissues against oxidation [3]. Other phytochemical compounds of saffron also include a wide range of flavonoids (mainly kaempferol derivatives), phenolic acids (hydroxycinnamic and hydroxybenzoic acids being the most representative) and phytosterols [4].

Owing to the presence of all these bioactive compounds, several therapeutic effects have been attributed to saffron, including cardiovascular disease prevention, insulin resistance, gastric disorders, depression, insomnia, premenstrual syndrome, anxiety, and neurodegenerative disorders, as well as bearing learning and memory-enhancing effects [3,4,5]. In the last decades studies demonstrating the anticancer and antiproliferative properties of saffron and its extracts have been published [6,7,8]. Among the three main saffron bioactive compounds, crocin has revealed to contain the most effective anticancer activity [9,10], and it has been demonstrated that crocin possesses antiproliferative effects on several cell lines derived from most types of human cancers [7], operating through several molecular mechanisms [7,9].

Although there are many studies regarding saffron addition to cheese infused [11,12,13], very limited works are reported in the literature concerning the characterization of saffron bioactive molecules in cheese, most of them regarding the determination of the volatile profile, including safranal [14,15], and only one regarding crocins and picrocrocin evaluation in cheese [16]. Furthermore, the antiproliferative effects of saffron have been mainly evaluated in pure saffron components [17,18,19,20] or its extracts [21,22,23], while there is a lack of studies concerning evaluation of the antiproliferative effect of saffron extract within a food matrix or in a complex mixture, additionally, no evaluation of the influence of the food chemical environment on saffron’s antiproliferative action has been reported.

Considering all these aspects, the objectives of this study were: (i) to characterize saffron bioactive molecules (crocins, picrocrocin and safranal) in cow and ewe cheeses made with saffron through a UHPLC (Ultra High Performance Liquid Chromatography) analytical method; (ii) to evaluate the in vitro antiproliferative effects of cheese extracts containing saffron bioactive compounds on different cellular lines, CaCo2 (colon cancer), HeLa (cervix carcinoma) and MDA-MB-231 (breast cancer); (iii) to evaluate any influence of the cheese matrix in modulating the antiproliferative effects of saffron’s bioactive compounds.

## 2. Results and Discussion

### 2.1. Saffron Bioactive Molecules in Cheese

**Figure 1:**
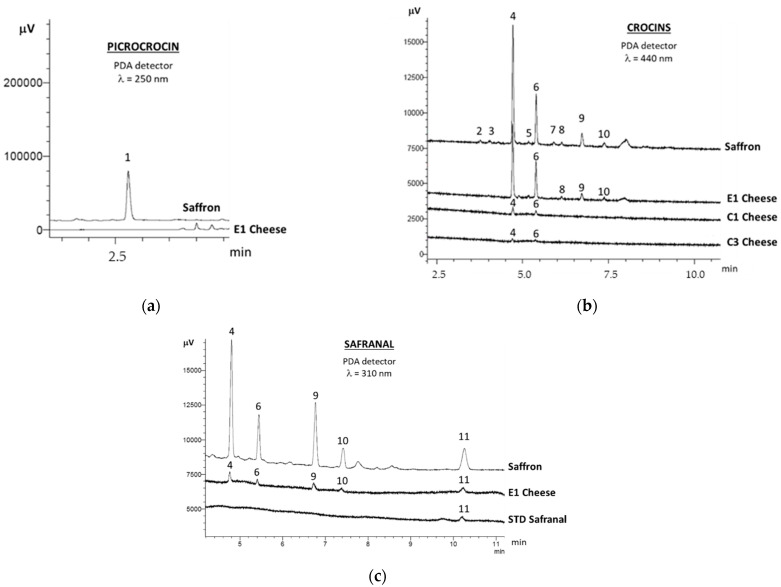
(**a**) UHPLC chromatograms of saffron and ewe cheese (E1) extracts detected at 250 nm; (**b**) UHPLC chromatograms of saffron, ewe (E1) and cow cheeses (C1, C3) extracts detected at 440 nm; (**c**) UHPLC chromatograms of saffron and ewe cheese (E1) extracts, together with a safranal standard (32 μg/L), detected at 310 nm: picrocrocin (peak 1); *trans*-crocins (peaks 2, 3, 5, 7, 8); *trans*-4-GG (peak 4); *trans*-3-gG (peak 6); *cis*-crocins (peaks 9, 10); safranal (peak 11).

Figure 1 shows UHPLC chromatograms of saffron bioactive molecules, picrocrocin, crocins and safranal, recorded at 250, 440 and 310 nm, respectively.

**Figure 2:**
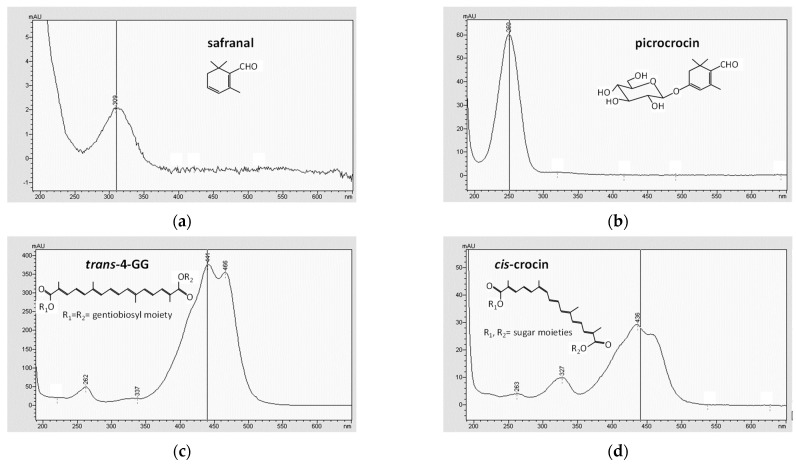
UV–Vis absorption spectra and chemical structures of saffron bioactive compounds: (**a**) safranal, (**b**) picrocrocin, (**c**) *trans*-4-GG, and (**d**) *cis*-crocin. All analytes were dissolved in methanol:water (80:20, *v*/*v*).

Picrocrocin (peak 1 in Figure 1a) was identified according to retention time of the standard and its UV spectrum [16], as reported in Figure 2. However, this molecule, to which saffron taste is related, was observed only in saffron extract and not in cheese extracts, confirming previous data reported in the literature according to which the lack of picrocrocin in the hydroalcoholic extract of cheese is probably due to its degradation during cheese making [16].

Regarding crocins, *trans*-4-GG, was the most abundant crocin observed in all cheese samples (peak 4 in Figure 1b), ranging between 45.4 and 69.8% of the total content. It was followed by another *trans*-crocin (peak 6 in Figure 1b), tentatively assigned to *trans*-crocetin (β-d-glucosyl)-(β-d-gentibiosyl) ester (*trans*-3-Gg), according to several studies which reported *trans*-4-GG and *trans*-3-Gg as the two main crocins present in saffron hydroalcoholic extracts [24,25,26]. *Trans*-3-Gg ranged between 26.3 and 43.5% of the total crocins content. Two ewe cheeses (E1 in Figure 1b,c, and E3 data not shown), as well as saffron (Figure 1b,c), also showed the presence of *cis*-crocins in their chromatographic profile (peaks 9–10 in Figure 1), while two cheese samples (C2 and E2, data not shown) showed only traces of the *cis*-crocin labelled as peak 9 in Figure 1. The recognition of *trans* and *cis* crocin isomers in the chromatographic profile of cheese was possible thanks to differences in their absorption spectra (see Figure 2c,d): *trans*-crocins show two absorption bands in their UV–Vis spectrum (250–260 nm, and 400–500 nm), while the *cis* isomers have a further absorption band at around 325 nm. The presence of both *trans* and *cis*-crocins in cheeses made with saffron had been previously reported only by Ritota, et al. [16], who ascribed the content of *cis*-crocins in cheese to their natural presence in saffron added to cheese and to the *cis*-*trans* isomerization reaction, promoted by light, which may occur during cheese making.

Table 1 reports the total crocins content (sum of all *cis* and *trans*-crocins) of the cheese samples, expressed as *trans*-4-GG content. Significant differences (*p* < 0.05) are probably due to the different amounts of added saffron and/or to the different cheesemaking technologies (saffron added in stigmas or powder, to milk or curd, before or after curd cooking, etc.). However, ewe cheeses generally showed higher crocin levels compared to cow cheeses.

The same analytical method for crocins determination was also used to characterize crocin-rich extracts from cheeses for their safranal content (Figure 1c). Safranal is a compound characterized by high volatility, hence UHPLC method would not be the suitable analytical technique for its determination since an underestimation of safranal content may occur. However, one of the aims of this work was to characterize saffron bioactive molecules in cheese through a single analytical method, and there are no data in the literature referring to safranal in cheese by UHPLC technique. As reported in Table 1, safranal was detected only in E1 ewe cheese, probably due to its high saffron content added during cheese making.

### 2.2. In Vitro Antiproliferative Assay of the Crocin-Rich Extracts

The antiproliferative effect of the crocin-rich extracts from cheeses was evaluated by in vitro study in three cellular models, cervix carcinoma (HeLa), colon cancer (CaCo2) and breast cancer. In order to exclude a possible interaction of estrogen receptor with the different molecules (fat residues) present in the extracts and to be able to compare the obtained results, a triple negative breast cancer line (MDA-MB-231) was used, which lacks estrogen and progesterone receptor expression, as well as human epidermal growth factor receptor 2 (HER2) amplification [27,28]. Total crocin contents of the cheese extracts were maintained constant (1 or 2 μM, according to the experimental needs) in order to evaluate any potential influence of the food matrix in modulating the antiproliferative effect of saffron bioactive compounds. Such crocin concentrations were obtained from data reported in the literature, and in particular from the work of Hire, et al. [29], who showed that crocin inhibited the assembly of microtubules, inducing a more significant antiproliferative effect in cancer cells (cervix and breast cancer cells), than non-cancerous fibroblast cells.

**Figure 3:**
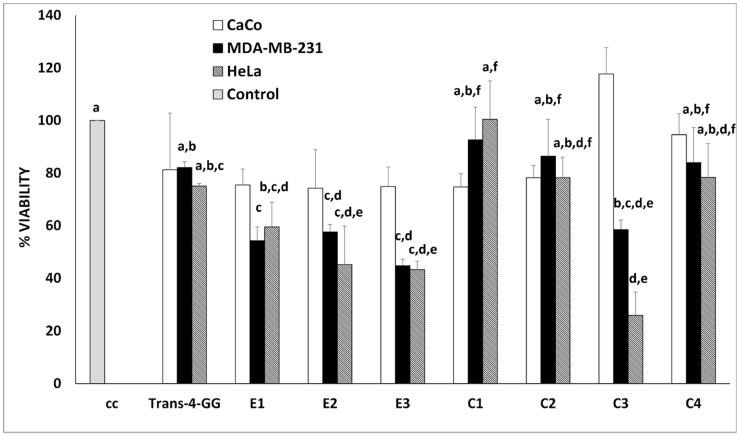
Antiproliferative effects (MTT assay) of a standard of *trans*-4-GG (2 μM) and of the crocin-rich extracts from cheese on CaCo2, MDA-MB-231 and HeLa cells models. Data are expressed as percentage of viability cell and presented as mean ± SE of three independent experiments, each conducted in triplicate. Different letters (a–f) mean significant differences (*p* < 0.05).

Figure 3 shows the results of MTT assay, where it can be observed that CaCo2 cellular model was not responsive to any experimental treatment, while significant effects were observed in HeLa and MDA-MB-231 cells.

Intestinal cells show greater resistance to external insults. In fact, some authors have shown that intestinal cell models are resistant to toxins [30], and exhibit a minor sensitivity at different treatments [31,32]. We hypothesize that the greater resistance of intestinal cell (CaCo2) could be due to the higher exposure to external insults compared to other tissues. Abdullaev Jafarova, et al. [33] had already observed an inhibitory effect of saffron extracts on the colony formation of some malignant cellular lines, reporting a major sensitivity of cervix carcinoma cells (HeLa) compared to colon carcinoma cells (SW480), 82% and 63%, respectively. Breast cancer cells (MDA-MB-231,) also showed a proliferation pattern similar to HeLa cells, although the significant antiproliferative effect in some treatments was less efficient. A similar result was observed by Gezici [34], who reported a greater inhibitory effect of saffron extracts on HeLa cells compared to epithelial tumor cell lines A549 and MCF, respectively, lung and breast cancer cells.

Although the results obtained in this study highlight and corroborate the major susceptibility of HeLa and MDA-MB-231 cell model to the antiproliferative activity of the crocin-rich extracts compared to intestinal cell model (CaCo2), it is worthwhile noting that this is the first time that saffron bioactive molecules present in a food matrix (cheese extracts) are evaluated for their antiproliferative effects on cancer model cell lines.

While no statistical differences are present in CaCo2 cell lines, crocin-rich extracts from E1, E2, E3 and C3 cheeses showed a significant (*p* < 0.05) antiproliferative effect in HeLa and MDA-MB-231 cells (Figure 3). In particular, viability decrease was most evident in C3 treatment both in HeLa and MDA-MB-231 cells (~75% and 42% of cellular death, respectively).

The antiproliferative effect of the crocin-rich extracts from cheese is certainly due to saffron bioactive compounds, mainly crocins, but the potential influence of the chemical environment of food matrix on the crocin antiproliferative effect cannot be neglected since, with the same concentration of crocins in the cheese extracts, differences in the antiproliferative effects were observed.

The natural structure of whole food certainly plays an important role in the physiological activity of bioactive compounds present within the food, also known as the “matrix effect” [35,36,37]. The food matrix, in fact, interacts with specific compounds, providing functionalities and behaviors which are different from those exhibited by the isolate components or in free state. Some authors showed that differences among food matrices are largely responsible for the different health properties of products that share similar chemical composition [38,39]. Despite this fundamental aspect, free state nutrients are still used in most in vitro studies, with results that are not reproducible in a food or whole diet.

In this study, in order to understand if the food matrix can affect crocins antiproliferative activity, the crocin-rich extracts incubated on the cellular lines were better characterized.

### 2.3. Characterization of the Crocin-Rich Extracts: Residual Protein Nitrogen and Fat

The use of a methanol:water (80:20, *v*/*v*) mixture as solvent for the extraction of saffron bioactive molecules from cheese could have resulted in the extraction of small peptides and/or short-chain fatty acids, as well as polar lipids.

Biological activities of milk-derived peptides have been known for a long time [40] and among them some peptides exert cytomodulatory action which may inhibit the growth of cancer cells [41]. Regarding cheese, instead, the characterization of peptides with antiproliferative properties is still ongoing [42]. The literature demonstrated the antiproliferative effect of methanolic extracts from cheeses on human leukemia cell lines [43], the inhibitory growth activities of water-soluble peptides extract of Cheddar cheeses on different cellular cancer models [44,45,46], and the antiproliferative effect on CaCo2 exerted by Mozzarella di Bufala waste whey peptides [47].

Among milk fat compounds, specific fatty acids, such as butyric acid, conjugated linoleic acid (CLA), phospholipids and sphingolipids from milk globule membrane (MGM) are considered to exert potential anticancer activity [48]. Yasuda, et al. [49], for example, observed that methanolic extracts from goat cheeses with different lipid contents exerted antiproliferative activity against human leukemia cells, as well as isolates from cream and buttermilk which showed antiproliferative effects against colon cancer cells both in vitro and in vivo [50,51].

Therefore, the hydroalcoholic extracts from cheese incubated on the cellular lines were characterized for their residual N/protein and fat, together with the “normalized” crocin content, namely the exact concentration of crocins incubated on cells (Table 2), in order to evaluate the potential influence of the chemical environment (food matrix effect) on the crocin antiproliferative effect.

Among ewe cheeses, with equal crocin contents incubated on the cells, the highest antiproliferative effects (E2 = E3 > E1, as reported in Figure 3) were observed in the samples with both high residual N/protein and fat contents.

Cow cheeses followed the same trend observed for ewe cheeses: C3 sample, which showed the highest antiproliferative effect, also had the highest residual N/protein and fat contents.

Despite the few samples analyzed in this study, Pearson’s correlation analysis showed results worth reporting (Table 3): with increasing N/protein in the crocin-rich extracts, a higher antiproliferative effect on HeLa cells was observed in ewe cheeses (r = 0.961), while a lower influence was observed for cow samples (r = 0.675, rising to 0.686 by excluding C2 sample which had a double crocins content compared to other cow cheeses). A similar result was also observed on MDA-MB-231 cell proliferation, but only in cow cheeses. The residual fat content in the cheese extracts, instead, showed a smaller correlation with the antiproliferative effect on HeLa cells in ewe cheeses (r = 0.663) compared to cow samples (r = 0.796 or 0.790, with or without C2 cheese, respectively). This could be due to the presence of different small peptides and fats in the extracts according to milk origin, but also to cheese ripening, which plays an important role in the release and formation of new peptides and fats [52]: according to the presence of specific compounds, these could result in exerting different biological activities. Different inhibitory effects of water-soluble peptides (WSPs) extracts of Cheddar were observed by Rafiq, et al. [45] according to the milk’s origin, with WSPs from buffalo Cheddar exerting a higher inhibitory effect on lung cancer cell line (H-1299) compared to WSPs from cow milk Cheddar. Furthermore, the same authors [45] observed different viability of the cancer cells during ripening. A positive correlation between ripeness and antiproliferative activity of various cheeses was also observed against human leukemia cellular models [43,49]. Finally, as for the N/protein, a higher antiproliferative effect on MDA-MB-231 cells was observed with increases in residual fat, but only in the crocin-rich extracts of cow cheeses (r = 0.822).

It is worthwhile highlighting that among cow cheeses, treatment with C2 extract, even if bearing a higher crocin content compared to other cow cheese extracts, did not show any significant effect on HeLa and MDA-MB-231 cell viability. On the other hand, cells treated with C3 cheese extract showed higher antiproliferative effects, confirming that chemical environment plays an important role in modulating crocin’s antiproliferative effects. Furthermore, with equal crocin contents incubated on the cells compared to ewe cheeses, treatment with C2 sample showed no significant antiproliferative effect despite its higher residual N/protein and fat contents), supposing a dissimilar influence of the chemical environment according to milk origin.

Abdullaev Jafarova, et al. [33] observed a different inhibitory behavior of the various saffron components on the colony formation of HeLa cellular lines, with *trans*-crocin 3 (*trans*-3-Gg) resulting in having the highest inhibitory activity, followed by picrocrocin in acidic form, picrocrocin and *trans*-crocin 4 (*trans*-4-GG). Furthermore, gentiobiosyl residues have been revealed to play an important role on the cytotoxic effect of saffron against tumor cells [53]. For these reasons, *trans*-3-Gg/*trans*-4-GG ratio was calculated in each crocin-rich extract (Table 2). Unfortunately, the major or minor presence of *trans*-3-Gg compared to *trans*-4-GG does not seem to explain the different behavior in the antiproliferative activity of the extracts: on the contrary, the cheese extracts with the lowest *trans*-3-Gg/*trans*-4-GG ratio showed the highest antiproliferative effects both on HeLa and MDA-MB-231 cells. This result could be related to a higher antiproliferative effect of *trans*-4-GG compared to *trans*-3-Gg, or to a greater contribution of the chemical environment to the antiproliferative effects of the cheese extracts compared to crocins. It was surprising to observe that a high correlation (r = 0.83) between antiproliferative activity and *trans*-4-GG percentage was observed only in cow cheese, whose r value rose up to 0.98 excluding C2 sample, which had double the crocins content compared to other cow cheeses extracts. This result would confirm the important role of the cheese matrix compounds in modulating the antiproliferative effect of crocins.

## 3. Materials and Methods

### 3.1. Samples and Chemicals

Different Italian cheeses, characterized by saffron addition during cheese making, were purchased in local markets or directly from the farmers. The samples evaluated in this study were as follows:

- three ewe milk cheeses (E1–E3) produced in three different farms and differing for ripening time and saffron addition.
- four cow milk cheeses (C1–C4), produced in the same farm and differing for indoor or local pasture feeding and for ripening time.

The main characteristics, as well as the way to which saffron was added during cheese making, are reported in Table 4. All dairy products were sampled and grated according to the reference method [54]. Three samples of the same cheese batch were analyzed, each one in triplicate.

The farm producing cow cheeses also provided saffron used in the cheese making.

*Trans*-crocetin di-(β-d-gentibiosyl) ester (*trans*-4-GG) (68% purity), safranal (purity 88%) and acetonitrile (for HPLC, ≥99.9%) were purchased from Sigma (Sigma–Aldrich Co., St. Louis, MO, USA), methanol (special grade, RS—for UHPLC-MS) from Carlo Erba (Carlo Erba Reagents S.A.S, Val de Reuil, France), while water was purified through a ion exchange Milli-Q system (resistivity >18 mΩ; Millipore, Billerica, MA, USA).

### 3.2. Saffron Bioactive Molecules in Cheese

Extraction and quantification of saffron bioactive molecules from cheese, namely crocins and picrocrocin, were carried out according to the method of Ritota, et al. [16]. Briefly 2.5 g of the grated cheese were extracted with 10 mL of a methanol:water (80:20, *v*/*v*) solution by stirring in the dark, at room temperature, for 1 h. The sample was then centrifuged (3500 rpm for 5 min at 4 °C) and the supernatant fraction was collected. Extraction was repeated twice more in the same way and the supernatant fractions were collected and filled to the mark (30 mL) with the same extraction solution. The extract was filtered (0.2 µm) before the UHPLC analysis. Chromatographic determination was carried out on a Nexera UHPLC system (Shimadzu Corporation, Kyoto, Japan) equipped with two LC-30AD pumps, UV–Vis SPD-M20A photodiode array (PDA) detector and SIL 30-AC autosampler, using a Synergi Fusion-RP (100 × 2.1, 2.5 μm, Phenomenex Inc., Torrance, CA, USA) column, with a SecurityGuard™ ULTRA cartridge for C18 UHPLC (2 × 2.1 mm, Phenomenex Inc., Torrance, CA, USA). LabSolution Version 5.42 SP5 (Shimadzu Corporation, Kyoto, Japan) software was used for equipment control, data acquisition and processing. Crocins were monitored at 440 nm, and the total crocins content was expressed as mg *trans*-4-GG/100 g cheese, by means of an external calibration curve for *trans*-4-GG (68% purity), and supposing the same response factor for all *cis* and *trans*-crocins. Picrocrocin was monitored at 250 nm.

Safranal content was detected in the same hydroalcoholic extract using the same chromatographic conditions proposed by Ritota, et al. [16], with PDA detector at 310 nm. Safranal content, expressed as mg/100 g cheese, was quantified by means of an external calibration curve (32–4235 μg/L). The recovery on a spiked sample was 72%, the instrumental Limit of Detection (LOD) and Limit of Quantification (LOQ) were equal to 0.006 and 0.014 mg/L, respectively (corresponding to 8 and 17 μg/100 g cheese), and the inter-day relative standard deviation (n = 3) was 0.4%.

### 3.3. Cell Lines and Treatment

HeLa, MDA-MB-231 and CaCo2 cells were obtained from the American Tissue Culture Collection (Manassas, VA, USA). The cells were grown in DMEM medium (Euroclone, Milan, Italy) supplemented with 10% Fetal Bovine Serum (FBS, Sigma–Aldrich Co., St. Louis, MO, USA), 100 U/mL penicillin and 100 µg/mL streptomycin (Pen/Strep, Euroclone, Milan, Italy), 2 mM glutamine (Euroclone, Milan, Italy) and 1% non-essential amino acid (Merck, Darmstadt, Germany). The cells were maintained at 37 °C in a humidified atmosphere of 5% CO_2_/95% air. 40.000 cells were seeded onto 24-multiwell plates.

The hydroalcoholic extracts from cheeses, rich in crocins, were filtered (0.2 µm) and brought to dryness under nitrogen flow, then they were dissolved in DMSO and added to the culture medium for 24 h.

### 3.4. In Vitro Viability Assay (MTT Assay)

A total of 0.5 mg/mL of MTT reagent (Merck, Darmstadt, Germany) was added to each well and incubated for 3 h at 37 °C in the CO_2_ incubator. The MTT solution was then discarded, and 100 μL of isopropanol was added. The plates were placed on a shaker to solubilize the formation of purple crystal formazan. The absorbance was measured using a microplate reader (Tecan 200 infinite pro) at a wavelength of 570 nm.

### 3.5. Residual Protein Nitrogen and Fat in the Crocin-Rich Extracts

The residual fat content in the hydroalcoholic crocin-rich extracts were determined according to the Bligh and Dyer [55] method. The residual protein nitrogen was determined according to the Kjeldahl method, using a multiplication factor of 6.38 [56], prior total evaporation of the hydroalcoholic crocin-rich extract and redissolution in ultrapure water.

### 3.6. Statistics

PAST 2.17 software [57] was used to perform statistical analysis. Normal data distribution and homogeneity of variance were tested using Shapiro–Wilk’s test and Levene’s test, respectively. One-way Analysis of Variance (ANOVA), followed by a Tukey’s Post Hoc with Bonferroni correction, was applied to evaluate significant differences (*p* < 0.05) among the mean values. In case of non-normal distribution and non-homogeneity of variance, Friedman and Kruskal–Wallis tests, respectively, were applied to test significant differences (*p* < 0.05) among data.

## 4. Conclusions

In this study, the main bioactive compounds of saffron (crocins, picrocrocin and safranal) were evaluated for the first time in cow and ewe cheeses made with saffron by a single UHPLC analytical method. Crocins were detected in all cheese samples, with levels varying according to the different cheese making process. Picrocrocin was not detected in cheese probably due to its degradation during cheese making, while safranal was detected only in the ewe cheese with the highest crocins content.

With the antiproliferative activity of saffron being known, and due to the lack of evidence of the antiproliferative effects of crocins within a food matrix or in a complex mixture, for the first time hydroalcoholic extracts from cheese containing saffron were tested for their antiproliferative activities against three cancer cellular models, CaCo2 (colon cancer), HeLa (cervix carcinoma) and MDA-MB-231 cells. No effect was observed on CaCo2 cells, probably due to a great resistance of intestinal cell to external insults, while HeLa and MDA-MB-231 cells were sensitive to treatment with crocin-rich extracts from cheese. The antiproliferative effects of the hydroalcoholic crocin-rich extracts of the studied cheeses could be ascribed to saffron’s bioactive compounds (mainly crocins), and to nitrogen and fat compounds present in the same extracts. However, a deeper characterization of the hydroalcoholic crocin-rich extracts could be helpful in better identifying the cheese compounds responsible, together with saffron crocins, for the antiproliferative effect in specific cell models (HeLa and MDA-MB-231, but not in CaCo2).

The chemical environment of the food matrix resulted in bearing a great influence in modulating antiproliferative effects of crocin: the crocin-rich extracts from cheese with both high residual N/protein and fat contents showed increased antiproliferative effects compared to pure crocin (*trans*-4-GG), probably due to milk origin and cheese ripening, both responsible for the presence of different type of fat compounds and small peptides.

The present study provides preliminary results concerning antiproliferative activity of extracts from cow and ewe cheeses made with saffron against cervix and breast carcinoma cell models. This could provide additional value to cheese made with saffron, both for farmers and consumers. Although these results are preliminary, additional studies are needed to investigate the antiproliferative effects of cheese extracts containing saffron in different cell lines and to research the molecular pathways involved. Finally, in order to evaluate the effect of bioactive compounds of saffron within food matrix, other studies should also be carried out on in vivo models (animals and/or in humans).

## Figures and Tables

**Table 1:** Characterization of saffron bioactive compounds in cheese (mg/100 g cheese): total crocins and safranal. Different superscript letters in the same column correspond to significantly different mean values (*p* < 0.05).

Sample	Total Crocins (mg/100 g Cheese)	Safranal (μg/100 g Cheese)
E1	30.57 ± 0.49 ^a^	57 ± 1
E2	2.87 ± 0.15 ^c^	n.d.
E3	10.02 ± 0.12 ^b^	n.d.
C1	0.83 ± 0.02 ^d^	n.d.
C2	1.49 ± 0.01 ^e^	n.d.
C3	0.54 ± 0.03 ^d^	n.d.
C4	1.36 ± 0.09 ^e^	n.d.

n.d. not detected, namely lower than safranal LOD. Different superscript letters (a–e) mean significant differences (*p* < 0.05).

**Table 2:** Main characterization of the crocin-rich extracts incubated on the cellular lines: total crocins (μM) incubated; residual protein nitrogen (mg/mL) and fat (mg/mL); *trans*-3-Gg/*trans*-4-GG ratio and *trans*-4-GG percentage.

Sample	Total Crocins (μM)	Protein N Residue (mg/mL)	Fat Residue (mg/mL)	*trans*-3-Gg/*trans*-4-GG	*trans*-4-GG (% on Total Crocins)
E1	2.00	2.52	0.35	0.5	57.3
E2	2.00	12.88	4.16	0.4	60.3
E3	2.00	10.98	1.53	0.7	46.4
C1	1.00	76.72	8.12	0.7	58.6
C2	2.00	77.66	7.72	0.5	56.1
C3	1.00	109.01	12.96	0.4	69.4
C4	1.00	44.58	5.36	0.6	63.8

**Table 3:** Pearson’s correlation analysis of the crocin-rich extracts in ewe and cow cheeses.

Ewe Cheeses				
	Protein N residue	Fat residue	HeLa Proliferation	MDA-MB-231 Proliferation
Protein N residue	1			
Fat residue	0.844	1		
HeLa Proliferation	−0.961	−0.663	1	
MDA-MB-231 Proliferation	0.098	0.450	0.370	1
**Cow Cheeses**				
	Protein N residue	Fat residue	HeLa Proliferation	MDA-MB-231 Proliferation
Protein N residue	1			
Fat residue	0.970	1		
HeLa Proliferation	−0.675	−0.796	1	
MDA-MB-231 Proliferation	−0.688	−0.822	0.993	1

**Table 4:** Main characteristics of the cow and ewe cheese samples.

Sample	Cheese Variety	Milk	Ripening	Saffron Addition
E1	Pecorino-type (ewe)	Pasteurized	30–40 days	Powder added during cutting of the curd
E2	Pecorino-type (ewe)	N.A. ^1^	about 15 days	Stigmas added during cutting of the curd
E3	Pecorino-type (ewe)	Raw	10 months	Powder (previously suspended in milk) added after cutting of the curd
C1	Cow hard cheese	Raw partially skimmed	22 months	Powder added to milk, or to a mixture of whey and curd, before cutting of the curd
C2	Cow hard cheese	Raw partially skimmed	14 months	Powder added to milk, or to a mixture of whey and curd, before cutting of the curd
C3	Cow hard cheese	Raw partially skimmed	19 months	Powder added to milk, or to a mixture of whey and curd, before cutting of the curd
C4	Cow hard cheese	Raw partially skimmed	16 months	Powder added to milk, or to a mixture of whey and curd, before cutting of the curd

^1^ N.A.: Not Available.

## Data Availability

The data presented in this study are available on request from the corresponding author.

## Sample Availability

Samples of the compounds are not available from the authors.

## References

[1] Basker D., Negbi M. (1983). Uses of saffron. Econ. Bot..

[2] Negbi M. (1999). Saffron cultivation: Past, present and future prospects. Saffron.

[3] Melnyk J.P., Wang S., Marcone M.F. (2010). Chemical and biological properties of the world’s most expensive spice: Saffron. Food Res. Int..

[4] Abu-Izneid T., Rauf A., Khalil A.A., Olatunde A., Khalid A., Alhumaydhi F.A., Aljohani A.S., Sahab Uddin M., Heydari M., Khayrullin M. (2020). Nutritional and health beneficial properties of saffron (*Crocus sativus* L.): A comprehensive review. Crit. Rev. Food Sci. Nutr..

[5] Akowuah G.A., Htar T.T. (2014). Therapeutic properties of saffron and its chemical constituents. J. Natl. Prod..

[6] Fernández J.-A. (2006). Anticancer properties of saffron, Crocus sativus Linn. Adv. Phytomed..

[7] Hoshyar R., Mollaei H. (2017). A comprehensive review on anticancer mechanisms of the main carotenoid of saffron, crocin. J. Pharm. Pharmacol..

[8] Lambrianidou A., Koutsougianni F., Papapostolou I., Dimas K. (2021). Recent advances on the anticancer properties of saffron (*Crocus sativus* L.) and its major constituents. Molecules.

[9] Bathaie S.Z., Bolhasani A., Hoshyar R., Ranjbar B., Sabouni F., Moosavi-Movahedi A.-A. (2007). Interaction of saffron carotenoids as anticancer compounds with ctDNA, Oligo (dG. dC) 15, and Oligo (dA. dT) 15. DNA Cell Biol..

[10] Festuccia C., Mancini A., Gravina G.L., Scarsella L., Llorens S., Alonso G.L., Tatone C., Di Cesare E., Jannini E.A., Lenzi A. (2014). Antitumor effects of saffron-derived carotenoids in prostate cancer cell models. BioMed Res. Int..

[11] Aktypis A., Christodoulou E.D., Manolopoulou E., Georgala A., Daferera D., Polysiou M. (2018). Fresh ovine cheese supplemented with saffron (*Crocus sativus* L.): Impact on microbiological, physicochemical, antioxidant, color and sensory characteristics during storage. Small Rumin. Res..

[12] Carpino S., Rapisarda T., Belvedere G., Licitra G. (2008). Volatile fingerprint of Piacentinu cheese produced with different tools and type of saffron. Small Rumin. Res..

[13] Licón C., Carmona M., Molina A., Berruga M. (2012). Chemical, microbiological, textural, color, and sensory characteristics of pressed ewe milk cheeses with saffron (*Crocus sativus* L.) during ripening. J. Dairy Sci..

[14] Librán C.M., Licón C.C., Serrano-Díaz J., Carmona M., Berruga M.I. (2014). Safranal transference from ewe’s milk to cheese and whey and antifungal properties of fortified whey. Dairy Sci. Technol..

[15] Licón C.C., Carmona M., Berruga M.I. (2015). Volatile compounds in pressed ewes’ milk cheese with saffron spice (*Crocus sativus* L.). Int. J. Dairy Technol..

[16] Ritota M., Mattera M., Costanzo M.G.D., Manzi P. (2018). Evaluation of Crocins in Cheeses Made with Saffron by UHPLC. J. Braz. Chem. Soc..

[17] Aung H., Wang C., Ni M., Fishbein A., Mehendale S., Xie J., Shoyama A., Yuan C. (2007). Crocin from Crocus sativus possesses significant anti-proliferation effects on human colorectal cancer cells. Exp. Oncol..

[18] Chryssanthi D.G., Dedes P.G., Karamanos N.K., Cordopatis P., Lamari F.N. (2011). Crocetin inhibits invasiveness of MDA-MB-231 breast cancer cells via downregulation of matrix metalloproteinases. Planta Med..

[19] Dhar A., Mehta S., Dhar G., Dhar K., Banerjee S., Van Veldhuizen P., Campbell D.R., Banerjee S.K. (2009). Crocetin inhibits pancreatic cancer cell proliferation and tumor progression in a xenograft mouse model. Mol. Cancer Ther..

[20] Mousavi S.H., Moallem S.A., Mehri S., Shahsavand S., Nassirli H., Malaekeh-Nikouei B. (2011). Improvement of cytotoxic and apoptogenic properties of crocin in cancer cell lines by its nanoliposomal form. Pharm. Biol..

[21] Bajbouj K., Schulze-Luehrmann J., Diermeier S., Amin A., Schneider-Stock R. (2012). The anticancer effect of saffron in two p53 isogenic colorectal cancer cell lines. BMC Complement. Altern. Med..

[22] D’Alessandro A.M., Mancini A., Lizzi A.R., De Simone A., Marroccella C.E., Gravina G.L., Tatone C., Festuccia C. (2013). Crocus sativus stigma extract and its major constituent crocin possess significant antiproliferative properties against human prostate cancer. Nutr. Cancer.

[23] Samarghandian S., Boskabady M.H., Davoodi S. (2010). Use of in vitro assays to assess the potential antiproliferative and cytotoxic effects of saffron (*Crocus sativus* L.) in human lung cancer cell line. Pharm. Mag..

[24] Caballero-Ortega H., Pereda-Miranda R., Abdullaev F.I. (2007). HPLC quantification of major active components from 11 different saffron (*Crocus sativus* L.) sources. Food Chem..

[25] Masi E., Taiti C., Heimler D., Vignolini P., Romani A., Mancuso S. (2016). PTR-TOF-MS and HPLC analysis in the characterization of saffron (*Crocus sativus* L.) from Italy and Iran. Food Chem..

[26] Tarantilis P.A., Polissiou M.G. Chemical analysis and antitumor activity of natural and semi-natural carotenoids of saffron. Proceedings of the I International Symposium on Saffron Biology and Biotechnology 650.

[27] Chavez K.J., Garimella S.V., Lipkowitz S. (2010). Triple negative breast cancer cell lines: One tool in the search for better treatment of triple negative breast cancer. Breast Dis..

[28] Liu H., Zang C., Fenner M., Possinger K., Elstner E. (2003). PPARγ ligands and ATRA inhibit the invasion of human breast cancer cells in vitro. Breast Cancer Res. Treat..

[29] Hire R.R., Srivastava S., Davis M.B., Kumar Konreddy A., Panda D. (2017). Antiproliferative activity of crocin involves targeting of microtubules in breast cancer cells. Sci. Rep..

[30] Calvert T., Aidoo K., Candlish A., Fuat A.M. (2005). Comparison of in vitro cytotoxicity of Fusarium mycotoxins, deoxynivalenol, T-2 toxin and zearalenone on selected human epithelial cell lines. Mycopathologia.

[31] William-Faltaos S., Rouillard D., Lechat P., Bastian G. (2006). Cell cycle arrest and apoptosis induced by oxaliplatin (L-OHP) on four human cancer cell lines. Anticancer Res..

[32] Zhou C., Tabb M.M., Sadatrafiei A., Grün F., Blumberg B. (2004). Tocotrienols activate the steroid and xenobiotic receptor, SXR, and selectively regulate expression of its target genes. Drug Metabol. Dispos..

[33] Abdullaev Jafarova F., Caballero Ortega H., Riverón Negrete L., Pereda Miranda R., Rivera Luna R., Hernández J.M., Pérez López I., Espinosa Aguirre J.J. (2002). Evaluación in vitro del potencial quimiopreventivo del azafrán. Rev. Investig. Clín..

[34] Gezici S. (2019). Comparative anticancer activity analysis of saffron extracts and a principle component, crocetin for prevention and treatment of human malignancies. J. Food Sci. Technol..

[35] Givens D.I. (2017). Saturated fats, dairy foods and health: A curious paradox?. Nutr. Bull..

[36] Lecerf J.-M., Legrand P. (2015). Les effets des nutriments dépendent-ils des aliments qui les portent? L’effet matrice. Cah. Nutr. Diététique.

[37] Zou L., Liu W., Liu C., Xiao H., McClements D.J. (2015). Utilizing food matrix effects to enhance nutraceutical bioavailability: Increase of curcumin bioaccessibility using excipient emulsions. J. Agric. Food Chem..

[38] Capuano E., Oliviero T., van Boekel M.A. (2018). Modeling food matrix effects on chemical reactivity: Challenges and perspectives. Crit. Rev. Food Sci. Nutr..

[39] Fardet A. (2014). Food health potential is primarily due to its matrix structure, then nutrient composition: A new paradigm for food classification according to technological processes applied. J. Nutr. Health Food Eng..

[40] Mohanty D., Mohapatra S., Misra S., Sahu P. (2016). Milk derived bioactive peptides and their impact on human health–A review. Saudi J. Biol. Sci..

[41] Meisel H., FitzGerald R.J. (2003). Biofunctional peptides from milk proteins: Mineral binding and cytomodulatory effects. Curr. Pharm. Design.

[42] López-Expósito I., Miralles B., Amigo L., Hernández-Ledesma B. (2017). Health effects of cheese components with a focus on bioactive peptides. Fermented Foods in Health and Disease Prevention.

[43] Yasuda S., Ohkura N., Suzuki K., Yamasaki M., Nishiyama K., Kobayashi H., Hoshi Y., Kadooka Y., Igoshi K. (2010). Effects of highly ripened cheeses on HL-60 human leukemia cells: Antiproliferative activity and induction of apoptotic DNA damage. J. Dairy Sci..

[44] Rafiq S., Gulzar N., Huma N., Hussain I., Murtaza M.S. (2020). Evaluation of anti-proliferative activity of Cheddar cheeses using colon adenocarcinoma (HCT-116) cell line. Int. J. Dairy Technol..

[45] Rafiq S., Huma N., Gulzar N., Murtaza M.A., Hussain I. (2018). Effect of cheddar cheese peptide extracts on growth inhibition, cell cycle arrest and apoptosis induction in human lung cancer (H-1299) cell line. Int. J. Dairy Technol..

[46] Rafiq S., Huma N., Rakariyatham K., Hussain I., Gulzar N., Hayat I. (2018). Anti-inflammatory and anticancer activities of water-soluble peptide extracts of buffalo and cow milk Cheddar cheeses. Int. J. Dairy Technol..

[47] De Simone C., Picariello G., Mamone G., Stiuso P., Dicitore A., Vanacore D., Chianese L., Addeo F., Ferranti P. (2009). Characterisation and cytomodulatory properties of peptides from Mozzarella di Bufala Campana cheese whey. J. Pept. Sci..

[48] Rodríguez-Alcalá L.M., Castro-Gómez M.P., Pimentel L.L., Fontecha J. (2017). Milk fat components with potential anticancer activity—A review. Biosci. Rep..

[49] Yasuda S., Kuwata H., Kawamoto K., Shirakawa J., Atobe S., Hoshi Y., Yamasaki M., Nishiyama K., Tachibana H., Yamada K. (2012). Effect of highly lipolyzed goat cheese on HL-60 human leukemia cells: Antiproliferative activity and induction of apoptotic DNA damage. J. Dairy Sci..

[50] Snow D.R., Jimenez-Flores R., Ward R.E., Cambell J., Young M.J., Nemere I., Hintze K.J. (2010). Dietary milk fat globule membrane reduces the incidence of aberrant crypt foci in Fischer-344 rats. J. Agric. Food Chem..

[51] Zanabria R., Tellez A.M., Griffiths M., Corredig M. (2013). Milk fat globule membrane isolate induces apoptosis in HT-29 human colon cancer cells. Food Funct..

[52] Santiago-López L., Aguilar-Toalá J.E., Hernández-Mendoza A., Vallejo-Cordoba B., Liceaga A.M., González-Córdova A.F. (2018). Invited review: Bioactive compounds produced during cheese ripening and health effects associated with aged cheese consumption. J. Dairy Sci..

[53] Bathaie S.Z., Mousavi S.Z. (2010). New applications and mechanisms of action of saffron and its important ingredients. Crit. Rev. Food Sci. Nutr..

[54] (2008). Milk and Milk Products—Guidance on Sampling.

[55] Bligh E.G., Dyer W.J. (1959). A rapid method of total lipid extraction and purification. Can. J. Biochem. Physiol..

[56] GU (Gazzetta Ufficiale della Repubblica Italiana) (1986). Metodi Ufficiali di Analisi per i Formaggi.

[57] Hammer Ø., Harper D., Ryan P. (2001). PAST, v. 2.17c. Palaeontol. Electron..

